# Coenzyme Q_10_ Treatment Monitoring in Different Human Biological Samples

**DOI:** 10.3390/antiox9100979

**Published:** 2020-10-13

**Authors:** Abraham J. Paredes-Fuentes, Raquel Montero, Anna Codina, Cristina Jou, Guerau Fernández, Joan Maynou, Carlos Santos-Ocaña, Joan Riera, Plácido Navas, Franchek Drobnic, Rafael Artuch

**Affiliations:** 1 Clinical Biochemistry Department, Institut de Recerca Sant Joan de Déu, Passeig Sant Joan de Déu, 2, 08950 Esplugues de Llobregat, Barcelona, Spain; ajparedes@fsjd.org (A.J.P.-F.); rmontero@sjdhospitalbarcelona.org (R.M.); 2 Pathology Department, Institut de Recerca Sant Joan de Déu, Passeig Sant Joan de Déu, 2, 08950 Esplugues de Llobregat, Barcelona, Spain; acodina@fsjd.org (A.C.); cjou@sjdhospitalbarcelona.org (C.J.); 3 CIBER de Enfermedades Raras (CIBERER), Instituto de Salud Carlos III, Calle Monforte de Lemos, 3-5, 28029 Madrid, Spain; csanoca@upo.es (C.S.-O.); pnavas@upo.es (P.N.); 4 Molecular Genetics Department, Institut de Recerca Sant Joan de Déu, Passeig Sant Joan de Déu, 2, 08950 Esplugues de Llobregat, Barcelona, Spain; gfernandezi@sjdhospitalbarcelona.org (G.F.); jmaynou@sjdhospitalbarcelona.org (J.M.); 5 Centro Andaluz de Biología del Desarrollo, Universidad Pablo de Olavide-CSIC-JA, Carretera de Utrera km 1, 41013 Sevilla, Spain; 6 Sport Nutrition and Physiology Department, Olympic Training Center, CAR-GIRSANE, Avinguda de l’Alcalde Barnils, 3, 08173 Sant Cugat del Vallés, Barcelona, Spain; jriera@car.edu (J.R.); docdrobnic@gmail.com (F.D.)

**Keywords:** coenzyme Q_10_, antioxidants, supplementation, treatment monitoring, skeletal muscle, urine, platelets, blood mononuclear cells, plasma

## Abstract

Coenzyme Q_10_ (CoQ) treatment monitoring is a matter of debate since CoQ distribution from plasma to blood cells and tissues is not fully understood. We aimed to analyze the CoQ levels in a wide set of human biological samples (plasma, blood mononuclear cells (BMCs), platelets, urinary cells, and skeletal muscle) from a group of 11 healthy male runners before and after CoQ supplementation. The CoQ content in the different samples was analyzed by HPLC coupled to electrochemical detection. No significant differences were observed in the CoQ levels measured in the BMCs, platelets, and urine after the one-month treatment period. Plasma CoQ (expressed in absolute values and values relative to total cholesterol) significantly increased after CoQ supplementation (*p* = 0.003 in both cases), and the increase in CoQ in muscle approached significance (*p* = 0.074). CoQ levels were increased in the plasma of all supplemented subjects, and muscle CoQ levels were increased in 8 out of 10 supplemented subjects. In conclusion, the analysis of CoQ in plasma samples seems to be the best surrogate biomarker for CoQ treatment monitoring. Moreover, oral CoQ administration was effective for increasing muscle CoQ concentrations in most subjects.

## 1. Introduction

Coenzyme Q_10_ (CoQ) is a lipid present in cell membranes, and it is ubiquitously synthesized in organs. CoQ is transported in the blood (serum) by cholesterol lipoproteins, and approximately 20% of total blood CoQ comes from dietary sources [1]. CoQ is composed of a benzoquinone ring and a polyisoprenoid side chain comprising ten units in humans. CoQ is involved in several biological functions, such as electron transport in the mitochondrial respiratory chain and ATP production inside the mitochondria. CoQ also plays key antioxidant functions in cell membranes and circulating cholesterol, protecting them from harmful oxidative damage [2].

CoQ deficiency may be associated with diverse inherited or acquired pathological conditions, and it may be defined by the decrease in CoQ content in biological specimens (being blood, muscle, and cultured skin fibroblasts the most common samples analyzed) [3]. Genetic primary CoQ deficiencies are inherited metabolic disorders due to mutations in the genes encoding proteins of the CoQ biosynthetic pathway (*COQ* genes), which impair mitochondrial oxidative phosphorylation and cause clinically heterogeneous phenotypes [4]. Secondary CoQ deficiencies have been associated with a wide variety of conditions, such as mitochondrial and non-mitochondrial metabolic disorders, neurodegenerative diseases, aging-relative oxidative stress, diabetes, cancer, cardiovascular diseases, and statin treatment. Thus, the diagnosis of CoQ deficiency seems important since early treatment initiation with oral CoQ may change the natural history of patients with these diseases. Patients with some forms of CoQ deficiency have shown clinical improvement with oral CoQ supplementation [5].

At present, CoQ treatment monitoring is a matter of debate. CoQ distribution from the serum to cells and tissues after oral intake is not fully understood, and the best biological sample to monitor patients under CoQ supplementation is currently under discussion [6]. Furthermore, different CoQ formulations are available with potentially different bio availabilities, depending on whether the CoQ is in the oxidized or the reduced form and what excipients are used [7]. There is also no agreement regarding the recommended supplementation dose. Some studies have reported changes in CoQ concentrations, mainly in muscle, plasma, blood mononuclear cells (BMCs), and platelets, after treatment [8,9,10,11,12,13,14,15]. CoQ may also be analyzed in other samples, such as urine, cerebrospinal fluid (CSF), and fibroblasts [6].

With this background, we aimed to analyze the CoQ levels in a set of human biological samples (plasma, BMCs, platelets, urinary cells, and skeletal muscle) in a group of healthy male runners before and after CoQ supplementation. We assessed the following: (a) which sample was the most appropriate for CoQ treatment monitoring, and (b) whether CoQ levels in the muscle increased after CoQ supplementation, as this is one of the target organs of CoQ therapeutic treatment.

## 2. Materials and Methods

### 2.1. Participants

A group of 11 healthy male runners was recruited for the study. They agreed to avoid the use of vitamin/mineral supplements during the duration of the study. During this period, all subjects maintained their physical activity with no changes in their diet or lifestyle. The mean and standard deviation (SD) of the age, weight, and body mass index of the subjects were 54.6 ± 4.3 years old, 76.8 ± 8.3 kg, and 25.1 ± 2.7 kg/m^2^, respectively. These subjects were studied at baseline (group 1, PRE) and after one month of CoQ supplementation (group 2, POST). It was not possible to perform a muscle biopsy in one of the subjects after CoQ supplementation due to a muscular injury that was not related to the study.

### 2.2. CoQ Treatment Protocol

Subjects took a CoQ phytosome capsule (UBIQSOME^®^, Indena SpA, Milan, Italy) (oral administration), equivalent to 100 mg of ubiquinone (the oxidized form of CoQ), once a day for four weeks. CoQ administration was carried out together with a meal at noon.

### 2.3. Samples

All samples were collected during the fasting state at baseline and after the one-month follow-up period.

- Blood samples: Ethylenediamine tetraacetic acid (EDTA) blood samples were drawn to separate BMCs, platelets, and plasma. Platelet-rich plasma (PRP) was obtained by centrifugation (60× *g*, 15 min, room temperature), as previously reported [16]. The PRP was then centrifuged (965× *g*, 20 min, room temperature) to pellet the platelets. The platelet count was analyzed in an ADVIA 2120 hematology analyzer (Siemens Healthineers, Erlangen, Germany). For BMCs isolation, the Histopaque 1077 procedure was used, as previously reported [17]. The plasma samples were separated by centrifugation (1500× *g*, 10 min, 4 °C). All the blood-derived samples were stored at −80 °C until CoQ analysis.
- Urine samples: The first morning urine samples were collected in standard urine containers. After centrifugation (1500× *g*, 10 min, 4 °C), the urinary pellet was washed with 9 mg/mL saline solution and then centrifuged as before to remove urinary proteins. The urinary pellet was stored frozen at −80 °C until CoQ analysis. The details of this procedure are reported elsewhere [18].
- Muscle samples: skeletal muscle samples were collected from the *vastus lateralis* muscle by vacuum-assisted biopsy. The muscle was weighed and homogenized with cold SETH buffer in an ice bath. The mixture was then vortexed, sonicated, and transferred to a polypropylene tube, followed by vortexing for 2 min, sonication for 5 min, and centrifugation (1500× *g*, 10 min, 4 °C). The supernatant was frozen at −80 °C until CoQ analysis.

### 2.4. CoQ Determination

**Figure 1:**
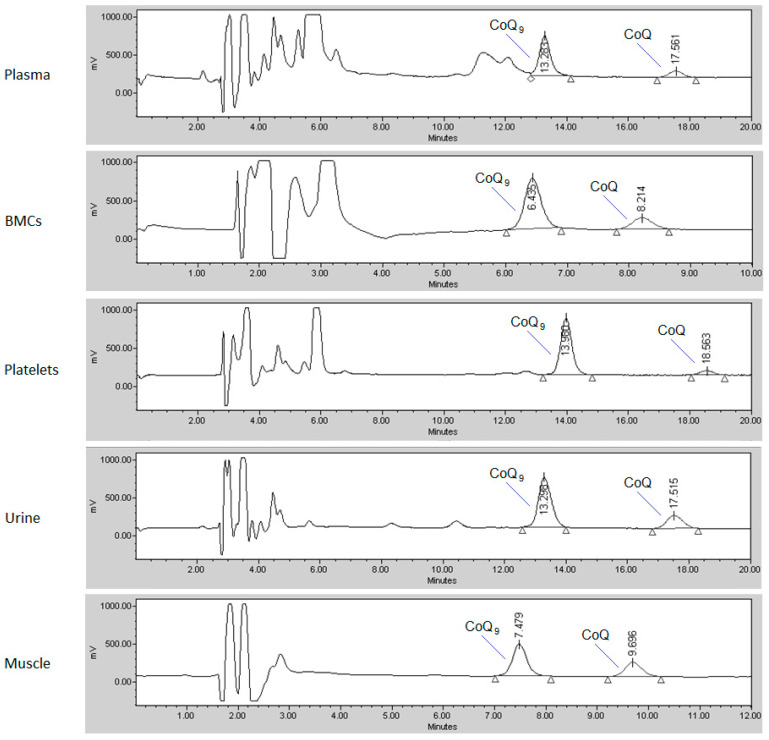
Typical chromatograms of coenzyme Q_10_ (CoQ) determination (HPLC with electrochemical detection) in the different biological samples: plasma, platelets, blood mononuclear cells (BMCs), urine, and muscle. Coenzyme Q_9_ (CoQ_9_) was used as the internal standard.

The CoQ content in the different biological samples was analyzed by HPLC (Waters, Milford, MA, USA) coupled to electrochemical detection (Coulochem II, ESA, Chelmsford, MA, USA), as previously reported [19]. Calibrators, controls, and biological samples were prepared in the same way. Internal standard (coenzyme Q_9_ [CoQ_9_] in ethanol) was added to all samples. After deproteinization, hexane was added to extract both CoQ_9_ and CoQ for 10 min by vortexing. After centrifugation at 1.500× *g* (10 min, 4 °C), the hexane phase was collected, filtered using a 0.22 µm filter, evaporated to dryness under a nitrogen stream, and re-dissolved in methanol/ethanol (60:40, *v*/*v*). The mobile phase consisted of 1.06 g/L lithium perchlorate in methanol/ethanol (60:40, *v*/*v*), and CoQ was separated in Nucleosil C-18 columns (Teknokroma, Sant Cugat del Vallés, Spain). For plasma, platelets, and urine CoQ separation, a column of 250 × 4.6 mm (length and internal diameter, respectively; particle size 5 µm) was used, since some naturally occurring interferences may elute close to CoQ_9_. For muscle and BMCs, a 150 mm length column was used, shortening the retention times. Typical chromatograms from the different specimens are shown in Figure 1. Once the CoQ was separated, it was quantified by electrochemical detection (the analytical cell was set to −600 mV and +600 mV; model 5010) using CoQ_9_ as the internal standard. The flow rate was 1 mL/min, and the injection volume was 50 µL (100 µL for urine samples). Since CoQ is related to cholesterol [20], total plasma cholesterol (Chol) values were analyzed by the automated cholesterol oxidase procedure in an Architect autoanalyzer (Abbott Laboratories, North Chicago, IL, USA). To estimate the cell content of the BMCs, urine, and muscle samples, the total protein concentration was determined by the Lowry method [21]. The CoQ values were normalized to the total protein concentration in the abovementioned specimens. In platelets, CoQ values were normalized to platelet count.

### 2.5. Ethical Issues

All subjects gave their informed consent for inclusion before they participated in the study. The study was conducted in accordance with the Declaration of Helsinki, and it was approved by the local Ethics Committee of the *Direcció General de l’Esport de la Generalitat de Catalunya* with the number 07/2018/CEICGC. The study was also registered at ClinicalTrials.gov with the identifier NCT03893864.

### 2.6. Statistical Analysis

The Wilcoxon test was applied to compare paired data between the baseline and one-month follow-up period in all the different variables. The Spearman correlation test was used to correlate plasma and muscle CoQ values. Statistical calculations were performed using IBM SPSS Statistics for Windows, version 25.0 (IBM Corp., Armonk, NY, USA).

## 3. Results

The biochemical results of healthy male runners recruited for CoQ supplementation are shown in Table 1. The data are expressed as the range, mean, and SD, together with previously reported reference values in the literature.

When we compared the subjects recruited for CoQ supplementation at baseline (group 1, PRE) and previously reported reference values, a good agreement was observed for CoQ values in plasma, BMCs, platelets, urine, and muscle samples. The muscle CoQ values in group 1 were similar to the reference ranges previously reported by our group and others [23,24].

**Figure 2:**
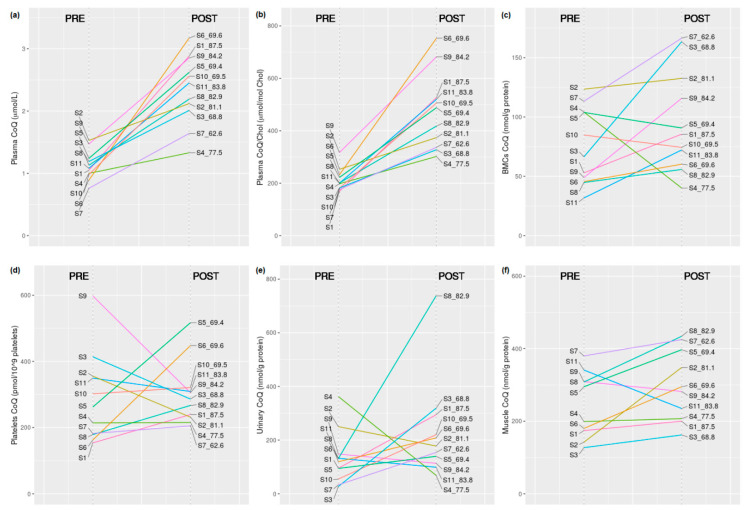
Graphic representation of the individual CoQ values obtained in the different biological specimens before (group 1, PRE) and after (group 2, POST) CoQ supplementation in the 11 participants: (**a**) plasma, (**b**) plasma CoQ/Chol ratio, (**c**) BMCs, (**d**) platelets, (**e**) urine, and (**f**) skeletal muscle. The body weight (kg) of each subject is indicated on the right side (POST).

**Figure 3:**
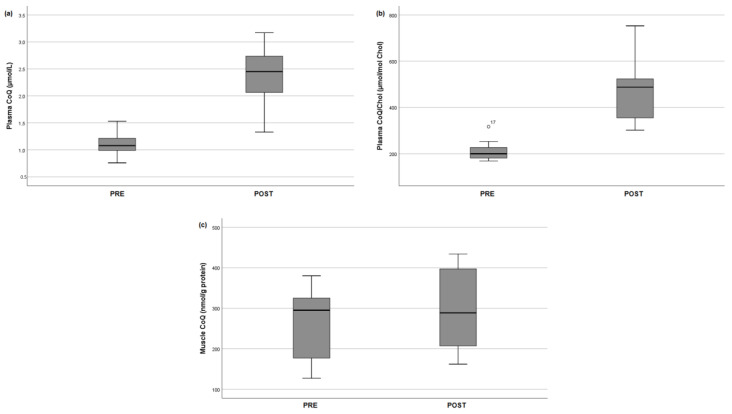
Box-plot representation of plasma CoQ values expressed as absolute values (**a**) and values relative to total cholesterol (**b**), and of muscle CoQ concentrations (**c**) before (PRE) and after (POST) CoQ supplementation. The length of the boxes indicates the interquartile space (p25–p75), the horizontal line into the box represents the median (p50), and the circle indicates an outlier value.

The Wilcoxon test was used to compare the CoQ status in the different samples before and after CoQ supplementation. No significant differences were observed in the CoQ levels measured in the BMCs, platelets, and urine (Figure 2). The plasma CoQ levels expressed in absolute values and relative to total cholesterol significantly increased after CoQ supplementation (*p* = 0.003 in both cases), and the increase in CoQ in skeletal muscle after CoQ supplementation approached significance (*p* = 0.074) (Table 1, Figure 2 and Figure 3). No correlation was observed between plasma and muscle CoQ values. After the supplementation period, the plasma CoQ concentration was increased in all the treated subjects, and the muscle CoQ levels were increased in 8 out of the 10 treated subjects (Figure 2 and Figure 3). Since the subjects’ weights ranged from 62.5 to 87.5 kg and all of them received the same amount of CoQ, the doses ranged from 1.14 to 1.60 mg/kg body weight (mean 1.33, SD 0.15). The two subjects who did not show increased muscle CoQ values weighed 83.8 and 84.2 kg (the second and the third in order of weight).

## 4. Discussion

CoQ treatment seems important, considering the key role of CoQ in vital biological processes. In this work, we analyzed several samples to assess which one is the most suitable to monitor during CoQ treatment and also to determine if CoQ supplementation increases CoQ levels in skeletal muscle. To the best of our knowledge, no studies have assessed CoQ status after supplementation in the wide array of biological samples studied here. The dosage employed is widely used in different food supplements [25].

Regarding the quantitative CoQ values in the different samples (Table 1), the concentration observed at baseline for group 1 was in agreement with our previous reference values and those reported by other authors [15,18,20,22,23,24].

When we compared the subjects before and after CoQ supplementation, no differences were observed in the BMCs, platelets, and urine CoQ values. The BMCs CoQ data are controversial: While Arias et al. found that lymphocyte CoQ concentrations were not significantly increased after CoQ supplementation at 100 mg/day for one week [11], other authors did find increased levels of CoQ in lymphocytes after CoQ supplementation over the same period and doses [13]; at higher doses (200 mg/day), increased CoQ in BMCs was demonstrated [12]. Regarding platelets, similar controversial results have been reported. We observed a slight but not significant increase in the mean platelet CoQ values after the supplementation period, although 4 out of 11 supplemented runners did not show any increase in CoQ. Niklowitz et al. reported that CoQ supplementation significantly increased the platelet CoQ levels after 14 days of supplementation (3 mg/kg body weight/day), with a further increase observed after 28 days, although this was not statistically significant [15]. Miles and coworkers reported similar results in the platelet total CoQ concentration after supplementation (5 mg/kg body weight, 28 days) [14]. In any case, in the abovementioned studies, the oral CoQ doses relative to body weight were moderately higher than those reported here. No previous studies have reported the use of urine CoQ levels for treatment monitoring purposes [18]. The involvement of the kidney in mitochondrial diseases and CoQ deficiencies strongly suggests that this non-invasive sample would be useful for treatment monitoring purposes as a surrogate biomarker of kidney CoQ status, considering that CoQ treatment regimens for mitochondrial disease patients (which can reach up to 30 mg/kg body weight/day) are remarkably higher than the doses employed in our study. In summary, these samples (BMCs, platelets, and urine) do not seem adequate for CoQ treatment monitoring, at least at these CoQ doses. A plausible explanation for this is that the CoQ status in BMCs, platelets, or urine probably displays a high biological variation. Moreover, the extensive preanalytical preparation required for cell isolation may also explain such variations, especially when compared with the direct measurement of plasma CoQ content.

Plasma was the best sample for monitoring, either analyzing total CoQ values or values relative to Chol, a molecule closely related to CoQ biosynthesis [20]. All 11 participants increased their plasma CoQ values after CoQ supplementation. This observation has been reported by several authors [8,12,15]. The main concern regarding plasma is that it only reflects the intestinal absorption and liver metabolism of supplemented CoQ but not the uptake of CoQ by tissues, which is, in fact, largely unknown in humans [26]. For this reason, we decided to measure muscle CoQ along with plasma CoQ.

Skeletal muscle is a tissue with high energy demands that is rich in mitochondria and CoQ. Most mitochondrial diseases have muscle as a target organ. Thus, knowing whether or not CoQ reaches the muscle is of paramount importance for the treatment of patients with mitochondrial diseases, inherited metabolic disorders, and neurogenetic diseases leading to CoQ deficiency. Although we did not observe significant differences in muscle before and after CoQ supplementation neither a correlation between plasma and muscle CoQ values, the truth is that 8 out of 10 supplemented subjects showed increases in their muscle CoQ values. Furthermore, the only two subjects whose muscle CoQ values did not increase after supplementation had two of the lowest CoQ doses in terms of their body weight (Figure 3). Plausible explanations for the lack of correlation between muscle and plasma CoQ would be the limited size of our series and that the biological factors modulating plasma and muscle CoQ status are different. Previous studies showed that oral supplementation (150 mg/day, 4 weeks) did not significantly alter the CoQ levels in skeletal muscle despite a significant elevation in the plasma CoQ concentration [9]. As observed here, Cooke and coworkers found that acute CoQ supplementation (200 mg/day) tended to increase the CoQ levels in muscle, although they did not observe significant changes following a 14-day supplementation period [8]. Considering that the CoQ doses relative to subject weights in previous reports were similar to those in our work, the differences observed could be explained by the differences in the CoQ formulations and excipients. In fact, considerable differences in the bioavailability of different CoQ formulations monitored in human plasma samples in an elderly population have recently been reported [25]. The formulation employed in this study (a phytosome capsule) is a mixture of phospholipids (lecithin) and CoQ, being CoQ placed among phospholipid molecules, which has been demonstrated to be safe and increase CoQ absorption in humans [27]. In any case, our findings strongly suggest that at higher CoQ doses, it is likely that all subjects would display an increase in the CoQ values in muscle.

## 5. Conclusions

In conclusion, the quantification of plasma CoQ values seems to be the best surrogate biomarker for oral CoQ treatment monitoring, since plasma CoQ increased in all cases in contrast to the variable responses observed in the other biological specimens. Under the conditions of our study, our results could potentially suggest that oral CoQ supplementation with this formulation was effective for increasing muscle CoQ concentrations in most subjects.

## Figures and Tables

**Table 1:** Biochemical results of subjects before (group 1, PRE) and after (group 2, POST) coenzyme Q_10_ (CoQ) supplementation. Data are expressed as range, average, and standard deviation, together with previously reported reference values in the literature.

	Plasma CoQ (µmol/L)	Plasma CoQ/Chol (µmol/mol Chol) and Chol (mmol/L)	BMCs CoQ (nmol/g Protein)	Platelets CoQ (pmol/10^9^ Platelets)	Urinary CoQ (nmol/g Protein)	Muscle CoQ (nmol/g Protein)
Group 1, PRE (*n* = 11)	0.76–1.53 1.11 (0.23)	169–317 212 (43.2) and 3.82–6.53 5.32 (0.82)	32–124 75 (32.4)	154–598 288 (135.5)	28–362 132 (98.2)	127–380 245 (90.7)
Group 2, POST (*n* = 11)	1.33–3.17 2.34 (0.55)	302–753 476 (145) and 4.18–6.13 5.02 (0.62)	40–166 96 (42.9)	205–517 304 (97.6)	69–738 230 (185)	162–434 299 (98.6) *
Reference values **	0.46–1.78 Ref. [22]	101–265 Ref. [22]	37–133 Ref. [23]	133–247 Ref. [15]	43–139 Ref. [18]	140–580 Ref. [23]

* The muscle biopsy of one subject could not be collected after CoQ supplementation. ** Reference values reported in the literature by other groups. A good agreement was observed with data from subjects at baseline (group 1) and also with our own reference values [18,20,24]. Abbreviations: BMCs, blood mononuclear cells; Chol, total plasma cholesterol; CoQ, coenzyme Q10.
